# Occurrence of Sweetpotato (*Ipomoea batatas*) Wilt and Surface Rot Disease and Determining Resistance of Selected Varieties to the Pathogen in Korea

**DOI:** 10.3390/plants9040497

**Published:** 2020-04-13

**Authors:** Narayan Chandra Paul, Won Park, Seungyong Lee, Mi Nam Chung, Hyeong-Un Lee, Jung-Wook Yang

**Affiliations:** 1 Bioenergy Crop Research Institute, National Institute of Crop Science, RDA, Muan 58545, Korea; ncpaul@korea.kr (N.C.P.); midaswon@korea.kr (W.P.); lsy8689@korea.kr (S.L.); minam@korea.kr (M.N.C.); leehu79@korea.kr (H.-U.L.); 2 Crop Cultivation and Environment Research Division, National Institute of Crop Science, RDA, Suwon 16613, Korea

**Keywords:** Fusarium wilt, *Fusarium oxysporum*, molecular phylogeny, surface rot, sweetpotato

## Abstract

Fusarium wilt and Fusarium surface rot caused by *Fusarium oxysporum* Schltdl are the major diseases of sweetpotato (*Ipomoea batatas*) and was surveyed in different locations (Cheongju, Heanam, Iksan, Icheon, Kimje, Nonsan, Yeoungam, and Yeoju) in Korea from 2015 to 2017 in the field, after harvesting and in storehouse. The wilt incidence in the early stage represented 17.9%, 5.9%, and 8.3% in 2015, 2016, and 2017, respectively. Samples were collected, and the causal organism was isolated on potato dextrose agar (PDA). Ten pure cultures were stored at the Sweetpotato Research Laboratory, Bioenergy Crop Research Institute, Muan, Korea. Morphological analysis, along with molecular phylogeny of the sequences of internal-transcribed spacer (ITS) and elongation factor 1-α (EF-1α) genes and their combined phylogenetic analysis, confirmed the isolates as the *Fusarium oxysporum* Schltdl. Pathogenicity tests were conducted on sweetpotato stems, and storage roots by artificially inoculation methods, and the most virulent isolate was selected as SPL18019. A rapid screening method on 21 selected varieties for resistant variety selection was applied on stems. The Pungwanmi was found resistant to Fusarium wilt, whereas Annobeni was the most susceptible. On the other hand, six varieties were used to test surface rot resistance, and Yulmi and Yesumi were resistant and susceptible, respectively, to Fusarium surface rot.

## 1. Introduction

The sweetpotato (*Ipomoea batatas* (L) Lam.) is one of the most important food crops in the world, is valuable in nutrition content, and possesses high levels of carbohydrate and dietary fiber [1]. It is a crop of high yield and a famine relief crop during hard times in Asia and Africa [2,3]. Recently, the sweetpotato become popular in Korea as a table food for breakfast and a diet food to reduce excess weight. People like to eat it using different ways of cooking such as in baked and steamed forms [1]. However, plant disease, as well as biotic and abiotic factors, plays a significant role in restricting high and quality yield worldwide [4]. A number of fungi produce disease in sweetpotato in different growth stages in the field and during storage. Among others, *Fusarium* comprises a diverse array of fungi and is common as a phytopathogenic fungus in different environmental conditions [5]. *Fusarium oxysporum* is responsible for severe damage to sweetpotato production by producing wilt disease in field and bed and surface rot on storage root, and it has caused destructive disease in sweetpotato producing countries around the world. The first symptom of wilt appears in the youngest leaves by changing the color to light yellow and yellowing between vines. Eventually, the entire plant collapses and dies. The pathogen can invade the fleshy roots and causes blackened ring about one-quarter inch below the surface, and sprouts produced from such sweetpotatoes are diseased. The disease remains in the storage roots and causes surface rot in the storage [6]. On the other hand, surface rot is characterized by its circular, somewhat sunken spots on the surface of the storage roots at their early stages. The spot areas gradually enlarge and become conspicuous in 6 to 8 weeks. The loss from surface rot is somewhat more than that of any other storage diseases [6]. In the cases of both of these two diseases, the pathogenic fungus *F. oxypsorum* is responsible. The forma specialis of the *Fusarium oxysporum* was determined and characterized on sweetpotato, and this is *F. oxysporum* f. sp. *batatas*, but the pathogen of the surface rot was described as *F. oxysporum* and identified previously [7,8,9,10]. These diseases are different; Fusarium wilt occurs in the field and surface rot in the storage. But the source of the pathogen might be the soil. The *F. oxysporum* is the main problem for quality productions as it could persist over a long period in the soil and infect storage roots [11].

Attempts have been made to eradicate the pathogen from various plants in different countries [12], but it was impossible to successfully eradicate it completely [13]. *F. oxysporum* is a ubiquitous, soil-borne asexual fungus that infects many plant species around the world [14]. This highly destructive pathogen is difficult to control or eradicate from the plant as well as from the soil. An alternative way has been searched to avoid or reduce the damage caused by this pathogen. Screening cultivars or varieties for Fusarium wilt is one of the effective ways to select the best resistant variety as well as to avoid the occurrence of the disease. Excellent control of this disease has been achieved by introducing high levels of resistance into new cultivars (USDA P.I. 153655, Tinian) [15,16]. The resistance has been quantitatively shown to be inherited. In Japan, resistant cultivar selection has been done and a resistant cultivar was selected (USDA P.I. 153909), which replaced the most serious disease with a less significant disease [17]. Screening has been conducted in different countries on sweetpotato and other plant species using conventional field trials or lab tests, which require significant time and space [18,19,20]. In Argentina, three cultivars collected from Argentina (Tucumana lisa), Brazil (Brasilera blanca) and the USA (Goldrush) were found resistant to the disease in sweetpotato [21]. In China, resistance was tested on 890 varieties from 1984 to 1987 and it was concluded that 41 varieties showed resistance, whereas 517 were highly susceptible [22].

Recently, a rapid seedling assay was conducted in sweetpotato in China by Yang et al. [20] to test the resistance of Fusarium wilt, and factors involving the resistant test were optimized, including conidial suspensions, incubation time, and temperatures. An improved method was described long ago by Hanna et al. in 1961 [16]. We followed these two methods with minor modifications. A fast and effective method was also developed for screening resistance to Fusarium wilt in banana (*Musa* spp.) [23]. The effectiveness and the degree of resistance and susceptibility may vary among years or soil type and also can be affected by other soil microbes [20,24]. Fusarium surface rot resistance was tested in some countries at a low scale, and it was observed that the small number of roots or cultivars or varieties are moderately resistant to this disease. Currently, no sweetpotato cultivars exist with complete resistance to surface rot disease. Long ago in 1926, Lauritzen [25] tested in the USA and observed that only a single cultivar showed resistance to surface rot disease. Fusarium wilt caused by *F. oxysporum* f. sp. *batatas* was reported from Korea [26], but to the best of our knowledge, the surface rot disease incidence of sweetpotato was not reported. Therefore, the present study aimed to investigate the disease occurrence of Fusarium wilt and surface rot, isolate and identify the causal organism, and evaluate several sweetpotato varieties for their resistance to Fusarium wilt and Fusarium surface rot by a rapid screening method. 

## 2. Results

### 2.1. Disease Incidence

**Figure 1:**
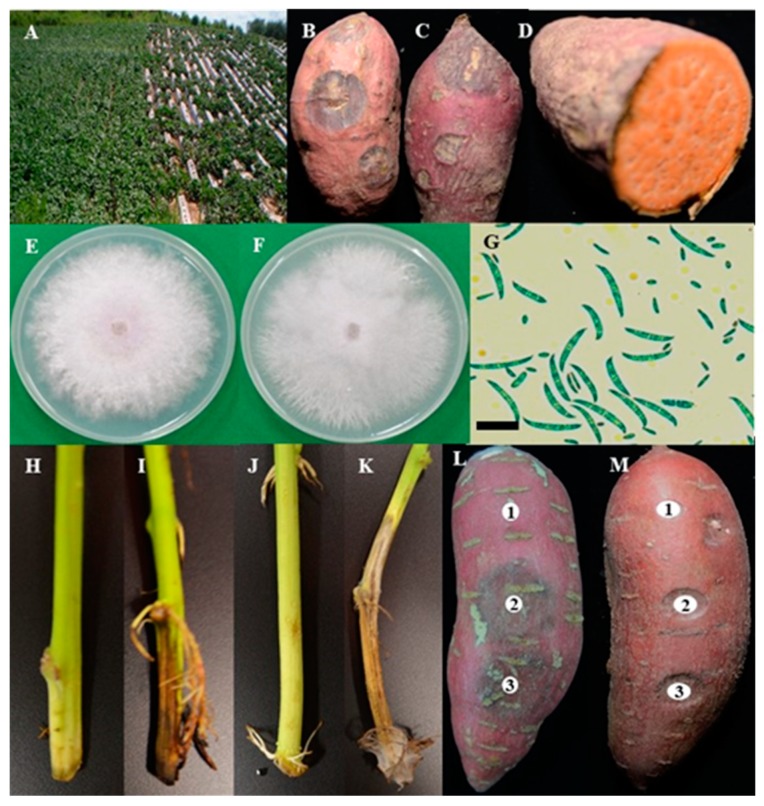
Diseased sweetpotato field with Fusarium wilt infection (**A**). Post-harvest diseased samples collected from storage (**B**–**D**). Colony morphology of the isolate SPL15020 on potato dextrose agar (PDA) (**E**) and malt extract agar (MEA) (**F**) incubated for 7 days at 25 °C. Macro- and microconidia (**G**). Pathogenicity on stems of Yulmi (**H**, control; **I**, inoculated) and Annobeni (**J**, control; **K**, inoculated). Pathogenicity on storage roots of Dahomi (**L**) and Hogammi (**M**) variety. Scale bars G = 20 μm, 1 = control and 2–3 = treated with the spore suspension (1 × 10^5^ spores/mL).

The disease incidence rate was highest in 2015 (17.9%), followed by 2017 (8.3%) and 2016 (5.9%) (Table 1). The disease rate was reduced due to following the proper, careful cultivation techniques and low rainfall. On the other hand, Fusarium surface rot usually infects through the wound during harvesting and post-harvest handling. Dried circular lesions on the surface of the sweetpotato storage roots were observed at the early stages of their infection, which became larger with age. The surface of the roots shrunk, but the lesions stayed reasonably close to the surface of the root. The tissue surrounding the lesion was dried, resulting in hard and mummified storage roots (Figure 1A−D).

### 2.2. Morphological Characterization

The conidial size slightly varies among isolates (Supplementary Table S1). The size of the macro- and microconidia of the isolate SPL15020 ranged from 10.8–35.3 × 2.4–5.2 μm to 3.1–7.7 × 1.4–3.4 μm, respectively (Figure 1G).

Macroconidia were slightly curved or straight, usually with three septations, and microconidia were elliptical to cylindrical with no septation (Supplementary Table S1). Morphologically, these isolates were identical to the previous descriptions of *F. oxysporum* [27].

### 2.3. Molecular Characterization

BLASTN analysis of the sequences indicated that the sequences from the present study matched well with the reliable reference sequences of *F. oxysporum* from GenBank. BLASTN search of all the sequences of the isolates showed 99–100% sequence similarity in internal-transcribed spacer (ITS) and EF-1α genes. Reliable reference neighboring sequences were copied and combined with these genes. A total of 36 combined sequences, including seven sequences from the present study, were analyzed for phylogenetic tree constructions. Sequences were submitted to GenBank for assigning accession numbers (Table 2).

**Figure 2:**
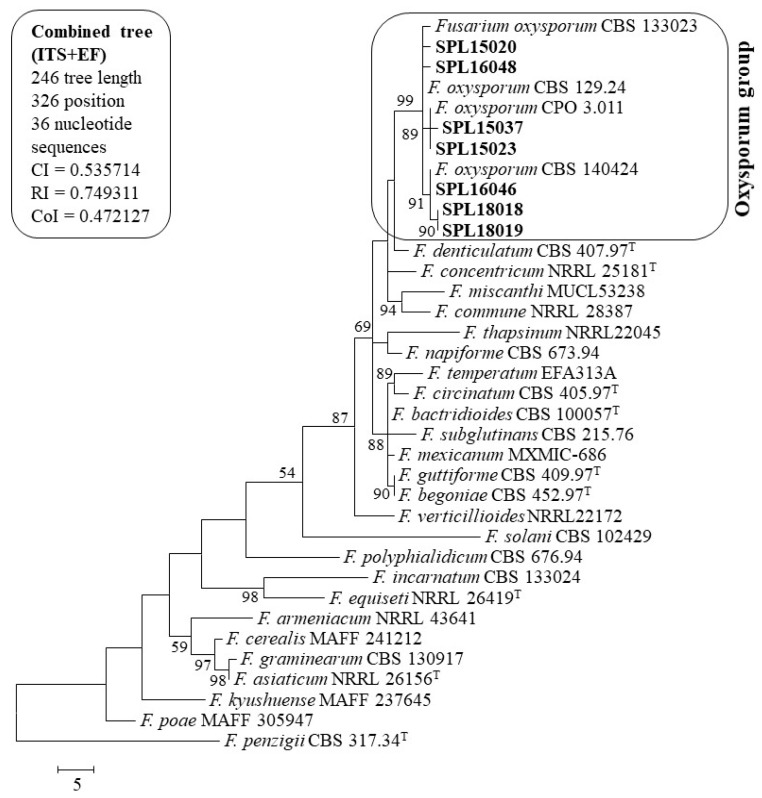
A parsimonious tree of the combined datasets of the internal-transcribed spacer (ITS) and EF-1α region of *Fusarium* and their related species from GenBank. Consistency, retention, and composite indexes are 0.600000, 0.793814, and 0.527731, respectively, for all sites and parsimony-informative sites. The percentage of replicate trees in which the associated taxa clustered together in the bootstrap test (1000 replicates) is shown next to the branches. Bootstrap values > 50% are indicated above branches. All positions containing gaps and missing data were eliminated. There were 326 positions in the final dataset. ‘T’ indicates type strain and present study isolates are in bold.

A maximum parsimony (MP) phylogenetic tree was constructed for the combined datasets of the ITS and EF-1α gene sequences. Furthermore, 1000 bootstrap replication was applied, and the percentage of replicate trees in which the associated taxa clustered together in the bootstrap test is shown next to the branches (Figure 2). Bootstrap values below 50% were deleted. Parsimony analysis (MP) of all sequences produced a single clade of the present study isolates (SPL15020, SPL15023, SPL15037, SPL16046, SPL16048, SPL18018, and SPL18019) with reliable references of *F. oxysporum* sequences and with a high bootstrap value. The isolates from wilt and surface rot diseases were not separated in the tree. Isolates SPL15020 (wilt pathogen) and SPL16048 (surface rot pathogen) were in a subgroup with *F. oxysporum* CBS 133023 and CBS 129.24. Isolates SPL15037 and SPL15023 were in the same subgroup with *F. oxysporum* CPO 3.011. The remaining isolates (SPL16048, SPL18018, and SPL18019) showed similarity with the reliable references of *F. oxysporum* CBS 140424. The sequences used in constructing phylogenetic trees were reliable and published, and some are type strains and came from authentic sources to the GenBank. Combined sequence analysis confirmed the present study isolates as *F. oxysporum* (Figure 2).

### 2.4. In Vitro Virulent Pathogen Screening and Resistant Variety Selection

**Figure 3:**
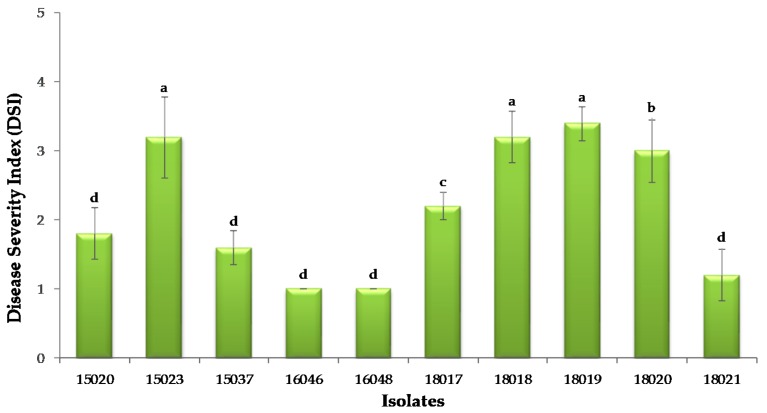
Virulence experiment conducted on stems of sweetpotato by 10 different isolates obtained in the present study. Disease severity index of the isolates is shown to choose the most virulent pathogenic isolate. Disease severity index (DSI) was measured by a 0–5 scale. 0 = seedlings grew normally without visible symptoms; 1 = ~5% browning and cracking in the vascular bundle; 2 = ~25% browning and cracking, 3 = ~25% browning and cracking in vascular bundle length and yellowing of the bottom leaves; 4 = browning of the entire vascular and discoloration of all the leaves; 5 = seedling collapsed and died. Different lowercase letters in the figure indicates the level of significance at *p* < 0.05 by Tukey’s test.

Ten *F. oxysporum* isolates were screened for virulent pathogen selection process on the known susceptible Annobeni variety. After 10 days of pathogen inoculation, disease symptoms appeared, and disease incidence was measured at 14 days. Overall, all the isolates showed disease incidence on sweetpotato stems in in vitro test. Disease severity in terms of disease index showed maximum by the isolate SPL18019 of all tested isolates, followed by SPL18018, SPL15023, and SPL18021, respectively (Figure 3). All the isolates showed disease incidence on a different scale. The virulence of the pathogen is different. Some isolates showed severe symptoms (e.g., SPL18019, SPL15023), and some showed minor disease symptoms (e.g., SPL16046, SPL18021). On the other hand, the length of the stem internal vascular bundle browning, a common symptom caused by *F. oxysporum* SPL18019, was also calculated, and it was observed that the highest lesion length was produced by the most virulent isolate (data not shown). Therefore, the most virulent pathogen *F. oxysporum* SPL18019 was selected for the variety selection for wilt resistance testing. The isolate SPL16048 was inoculated to the six sweetpotato storage roots for the pathogenicity test and variety selection for the Fusarium surface rot disease. Disease symptoms were observed in all six varieties tested, whereas the control remained asymptomatic (Figure 1L–H). The fungus was re-isolated from the lesions to fulfill Koch’s postulates and analyze the morphological characteristics, and conidial morphology was found as well as colony characteristics that were similar to the original.

**Figure 4:**
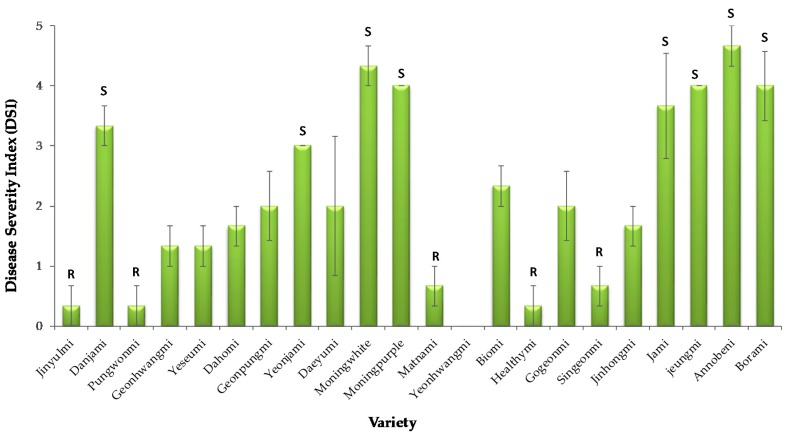
Effect of pathogen infection on the stems of 21 different sweetpotato varieties inoculated *F. oxysporum* SPL18019 spore suspensions with a concentration of 1 × 10^5^ spore/mL. Here, S indicates susceptible, and R denotes resistant variety. Disease severity index (DSI) is measured by a 0–5 scale. 0 = seedlings grew normally without visible symptoms; 1 = ~5% browning and cracking in vascular bundle length; 2 = ~25% browning and cracking, 3 = ~25% browning and cracking in vascular bundle length and yellowing of the bottom leaves; 4 = browning of the entire vascular and discoloration of all the leaves; 5 = seedling collapsed and died. DSI > 1 means resistant, 1–3 is termed moderate, and <3 means susceptible.

The spore suspensions of the isolate SPL18019 were inoculated in stems of 21 sweetpotato varieties, and almost all the varieties showed disease incidence on different scales except Yeonhwangmi. In the present scale explained in the methods section, six varieties showed resistance to Fusarium wilt disease caused by *F. oxysporum*, and the varieties were Jinyulmi, Pungwonmi, Matnami, Yeonhwangmi, Healthymi, and Singeonmi. The highly susceptible varieties were Annobeni, Morning white, Morning purple, Jeungmi, and Danjami. Other varieties were moderately resistant and moderately susceptible in sweetpotato (Figure 4). *Fusarium* spp. transfers from field to storage by wounding caused by improper handling. We did not focus on the resistance variety selection on a large number of varieties for Fusarium surface rot disease. We experimented on six popular varieties, and results revealed that Yesumi was the most susceptible one followed by Dahomi. On the other hand, Beniharuka and Yulmi were the most resistant variety followed by Pungwanmi and Hogammi (data not shown).

## 3. Discussion

**Figure 5:**
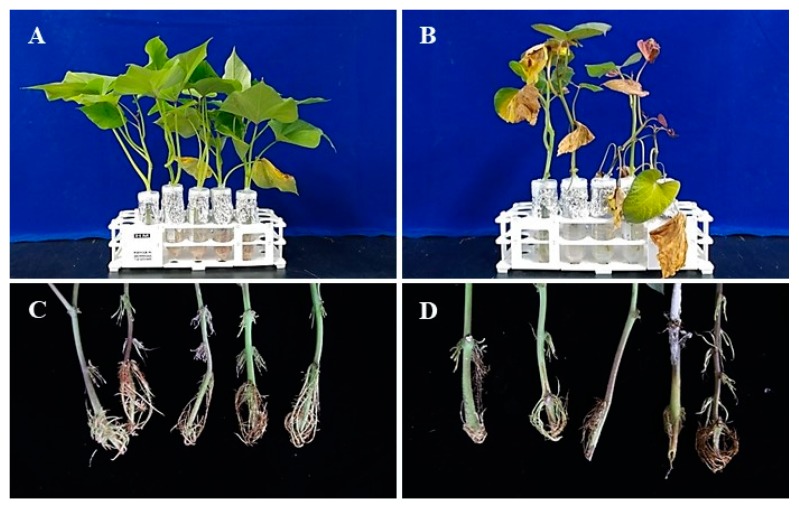
The disease reaction of the selection for evaluation of resistance to *F. oxysporum* SPL18019. Here, the photograph shows the control (**A**,**C**; no visible symptoms) and pathogen-infected (**B**, leaf yellowing; **D**, yellowing and cracking root tissues) of the Annobeni variety.

Fusarium wilt and surface rot diseases of sweetpotato and their causal organism were investigated in the present study, which applied an established rapid screening method [20] with minor modifications for resistant variety selection. We followed their optimized conditions of temperature, relative humidity, and concentration of spore suspensions, but each seedling was immersed in spore suspensions separately in a 50 mL falcon tube (Figure 5), while the previous report [20] explained that they immersed all the seedlings in the same bottle. Our explanation is that separate immersion could reduce the risk of contamination if there are any contaminated seedlings. The disease incidence was monitored on a yearly basis, and the variation in different years was observed. The reason behind the variation might be the high rainfall, which spreads *Fusarium* spores easily and quickly [28]. In 2015, the percent rainfall was higher compared to the other years examined. On the other hand, Fusarium diseases are common in storage all over the world. Sweetpotato storage roots are infected by a number of common *Fusarium* spp., and reported from different countries [29]. *Fusarium* spp. requires a wound to infect, and there are two common *Fusarium* spp. that severely infect sweetpotato—*F. oxysporum* and *F. solani*.

In the present study, we focused on investigating, isolating, and identifying the *F. oxysporum* pathogen. We collected samples mainly from wilt-infected field and then from surface-rot-infected storehouses in Korea. The fungus was isolated and preserved as well as identified through morphological and molecular methods. Diversity was observed among the isolates on the basis of a morphology study, which includes colony color, colony morphology, conidiation, size, and shapes of conidia. Mycelia were white, peach, pinkish-white, and brownish, but the shapes of conidia were slightly curved or sickle-shaped. All the conidia were elliptical to cylindrical without septation, and size varied slightly with isolates (Supplementary Table S1). In 2012, Joshi et al. [30] also explained the morphological differentiation among *F. oxysporum* isolates, that is, slight variations in conidial sizes were common among isolates, and the present study isolates matched well with the morphological description of *F. oxysporum*.

Accurate identification through morphological methods is always problematic. The molecular identification with multigene phylogeny could help to overcome the problem. We tried to identify them using two primer pairs of ITS rDNA and translation elongation factor (EF-1α) gene sequences. These gene sequences were the possible solution for *Fusarium* diagnostics along with morphology study [2,31]. Phylogenetically, isolates produced single clades, but there were subclades, which means there may have been race differentiation. Clark et al. [7] analyzed race differentiation of *F. oxysporum* in sweetpotato and tobacco based on the analysis of similarity coefficients on RAPD profiles and showed the discrete clusters of isolate representing different lineages and were distinguished from each other by forma specialis. Within a forma speciales, races are frequently distinguished by their specific pathogenicity to different cultivars. In the USA, sweetpotato germplasm resistance was screened by ‘race 0’ (Southeastern US) under the forma specialis *F. oxysporum* f. sp. *batatas* and ‘race 1’ from California, which causes wilt on cultivars resistant to ‘race 0’ [7,32]. Three races (1–3) of *F. oxysporum* f. sp. *Lycopersici* that caused tomato wilt were reported, which were classified on the basis of their virulence on tomato [32]. In the present study, we characterized the isolates up to the species level to find out whether there were any other species besides *F. oxysporum*, and the result revealed that there was only one species involved that showed these two diseases in sweetpotato.

We tested 10 isolates for preliminary screening on the Annobeni variety (Figure 5), commonly known as a susceptible variety in Korea. This part of the experiment was to confirm the pathogenicity and disease severity of the isolates to choose the most virulent pathogenic isolate by using the methods developed previously [16,22]. For quick, easy, and accurate screening of Fusarium wilt resistance, it was necessary to develop a new method or modify and adjust available methods. However, we applied the method to evaluate the resistance of 21 varieties and replicated it three times. The *F. oxysporum* concentration was adjusted to 1 × 10^5^ spores/mL, and stems were dipped in spore suspensions and incubated at 28 °C in an incubator [22]. After 10 days of incubation, disease symptoms were observed, and we counted the disease severity and made a disease index. As *Fusarium* needs a wound to penetrate [33], stems were cut just before applying the spore suspensions. The degree of wilting and vascular bundle yellowing and cracking varied with varieties. Our result revealed that most of the varieties were moderately susceptible to *F. oxysporum*, and few showed resistance. The previous experiment suggests that few sweetpotato varieties are resistant to Fusarium wilt disease [22]. An experiment on basil was conducted for resistance variety selection in Israel [34]. They selected resistant individuals, and resistant progenies, which exhibited remarkable resistance to Fusarium wilt and were used as a source for the production of a favorable commercial cultivar.

We tested the disease resistance variety selection on storage roots of six different varieties. As mentioned above, proper sanitation could reduce the infection in storage, so we could avoid the storage disease by reducing the disease in the field. Fusarium surface rot in all varieties did not show the same symptoms, but some variety showed resistance and some were moderately susceptible. The resistance of sweetpotato variety differs significantly in storability, so the major emphasis is given to storability in many regions in the world [8,35]. Besides this, resistance variety selection has been done to avoid surface rot disease. Similar observations were recorded in Bangladesh [36], China [20], India [37], Peru [38], and the USA [35]. In Korea, severe surface rot disease was observed in the Yesumi variety, Yulmi showed resistance to the disease, and the other varieties were resistant to moderately susceptible to the disease. The surface rot disease could be reduced by reducing the mechanical injury and proper management practices, including curing [39].

This method applied to test wilt resistance is fast and easy to apply and was conducted in the lab and in the greenhouse. With this method, a large population could be screened in a short period of time. In conclusion, we isolated a number of isolates from wilt and surface rot diseases identified as *F. oxysporum.* The modified rapid method applied has several advantages, including less time and space required, and the fact that the experiment can be conducted in the growth chamber of a lab. The method is applicable to select the resistant variety against *F. oxysporum*.

## 4. Materials and Methods

### 4.1. Disease Incidence

Disease incidence is the assessment of disease that is present in the field. It can be measured as the proportion of a plant community that is diseased (disease incidence) or as the proportion of plant area that is affected (disease severity). The occurrence of fusarium wilt disease was surveyed by eye estimation in 8 different locations (Cheongju, Heanam, Iksan, Icheon, Kimje, Nonsan, Youngam, and Yeoju) in Korea. Wilting symptoms, along with cracking of stems, was observed during May–June in 2015 to 2017. The presence of disease was monitored and noted in a laboratory notebook, and the land area cultivated was noted. After getting data from different locations, the disease incidence was calculated by the following formula explained by Teng and James in 2001 [40].

(1) $$I\;(\%)\;=\;\frac{\mathrm{ni}}{N}\times 100$$

where I = Disease incidence, ni = total number of affected plants, and N = total number of evaluated plants.

### 4.2. Pathogen Isolation and Culture Maintenance

Infected samples were collected from different locations from 2015 to 2017, washed under running tap water to remove the soil contaminants and debris, air-dried before isolating the causal pathogen. Washed and dried samples were surface-sterilized with 1% sodium hypochlorite for 5 min, followed by washing three times with sterile distilled water [41]. The surface-sterilized samples were allowed to dry on sterile kitchen towels in a laminar airflow chamber. Samples were then cut into small pieces and placed onto potato dextrose agar (PDA) supplemented with the antibiotic rifampicin (0.4 mg/mL, Sigma-Aldrich, Munich, Germany) to suppress bacterial growth. After incubation at 25 °C for 4 days, individual hyphal tips of the developing fungal colonies were removed and transferred onto PDA and further incubated for 5–10 days. *Fusarium-*like colonies were transferred to a new PDA Petri dish and cultured several times for culture purity. Ten isolates were then selected for next work, assigned identification numbers (SPL15020, SPL15023, SPL15037, SPL18017, SPL18018, SPL18019, SPL18020, and SPL18021 from wilt samples and SPL16046 and SPL16048 from surface rot samples), maintained on PDA slant tubes, and stored in 20% glycerol at −80 °C at the Bioenergy Crop Research Institute Fungal Herbarium, National Institute of Crop Science, Rural Development Administration, Muan, Korea.

### 4.3. Morphology

Fungal isolates were grown on potato dextrose agar (PDA) and malt extract agar (MEA) at 25 °C in the dark to facilitate the colony morphology, texture, color, and conidiation for microscopic examination. Conidiation was induced by culturing the colony (after scratched-off mycelium) at 12h–12h light–dark condition. Temporary slides were prepared for each isolate, mounted in lactophenol blue solution (Sigma-Aldrich, Munich, Germany), and examined under a light microscope (Zeiss, Oberkochen, Germany). In order to describe colony color, descriptions of aerial mycelium, conidial size, shape, and conidia color of the strains were observed using a BX50 microscope (Olympus, Tokyo, Japan) with Artcam 300 MI digital camera (Artray, Tokyo, Japan). Colors were named using “A Mycological Colour Chart” [42]. Morphological characteristics of the isolate were then compared with previous descriptions.

### 4.4. Molecular Phylogeny

The total genomic DNA of the isolates was extracted directly from the mycelia grown on PDA using the Solg Genomic DNA Prep kit (Solgent Co., Ltd., Daejeon, Korea) to confirm the identity of the fungi. Two gene regions, namely the internal-transcribed spacer (ITS) and elongation factor (EF-1α) were amplified using polymerase chain reaction (PCR). The gene regions were amplified with ITS5 (5′-GGA AGT AAA AGT CGT AAC AAG G-3′) and ITS4 (5′-TCC TCC GCT TAT TGA TAT GC-3′) [43] and EF1 (5′-ATG GGT AAG GAR GAC AAG AC-3′) and EF2 (5′-GGA RGT ACC AGT SAT CAT GTT-3′) [44]. The PCR amplification was carried out using a Bio-Rad PCR System T100 thermocycler (Bio-Rad Laboratories, Hercules, CA, USA) in a 20 μL reaction volume containing 5 μL 10× *e-Taq* reaction buffer, 1 μL 10 mM dNTP mix, 1 μL of each primer, 1 μL template DNA solution, 1.5 μL *Taq-*DNA polymerase, and sterilized distilled water. The PCR products were purified using the Accuprep PCR purification kit (Bioneer Corp., Daejeon, Korea) according to the manufacturer’s instructions. The PCR amplification was carried out under the following conditions: initial denaturation at 94 °C for 5 min, followed by 35 cycles of denaturation at 94 °C for 35 s and annealing at 55 °C for ITS, 56 °C for EF 1-α, and a final extension at 72 °C for 2 min. PCR products were sequenced by a commercial sequencing service provider (Macrogen, Daejeon, Korea) in both directions.

To identify genes with similar sequences, present sequences were searched with Basic Local Alignment Search Tool (BLASTN) searches using the National Center for Biotechnology Information (NCBI) database (http://www.ncbi.nlm.nih.gov). Closely related published and reliable sequences were obtained from GenBank (http://www.ncbi.nlm.nih.gov) for phylogenetic analysis. Sequences were aligned using ClustalX v.1.83 [45], and the sequence ends were manually trimmed to remove low-quality bases using BioEdit v. 5.0.9.1 program [46]. Maximum parsimony analysis was performed using MEGA 6.06 program [47] to construct phylogenetic trees.

### 4.5. Virulence, Pathogenicity, Disease Severity, and Resistant Variety Selection

Pathogenicity was performed with the ten isolates (SPL15020, SPL15023, SPL15037, SPL16046, SPL16048, SPL18017, SPL18018, SPL18019, SPL18020, and SPL18021) on a susceptible local sweetpotato variety, namely Annobeni. The isolates were grown on PDA for 7 days at 25 °C before inoculation, the mycelia were scratched off using a needle, and sporulation was induced under 12 h–12h light–dark conditions. After 2 days of culture, spores were collected and counted using a hemocytometer. Stems were surface-sterilized by dipping in 1% NaOCL for 5 min prior to washing with sterilized distilled water 3 times. After drying, the basal parts of the stems were dipped in spore suspensions (1 × 10^5^ spores/mL) with 5 replications and incubated at 28 °C in a growth chamber for three weeks in light–dark condition. After 10 days, disease symptoms appeared, and disease incidence was measured at 14 days. On the other hand, the isolate SPL16048 obtained from surface rot of sweetpotato were tested for pathogenicity on tubers of six different local varieties: Beniharuka, Dahomi, Hogammi, Pungwanmi, Yesumi, and Yulmi. Storage roots were surface-sterilized in 1% NaOCl for 10 min and then washed three times in sterile distilled water. Sweetpotato storage roots were inoculated by creating three wounds (6 mm in size) in each storage root, and inoculating two wounds with 20 μL of the fungal spore suspension at a concentration of 1 × 10^5^ spores/mL. The third wound was used as a control and inoculated with the same volume of sterilized distilled water. The storage roots were kept in moistened clean boxes and incubated at 28 °C. The pathogenicity experiment was conducted three times with three replications. After incubation for 3 weeks, the lesion length was observed and measured and the disease severity index was prepared [48].

After selecting the most virulent pathogen (SPL18019), disease-resistant variety selection experiment was conducted on 21 well-known sweetpotato varieties in Korea: Jinyulmi, Danjami, Pungwonmi, Geonhwangmi, Yesumi, Dahomi, Geonpungmi, Yeonjami, Daeyumi, Morning white, Morning purple, Matnami, Yeonhwangmi, Biomi, Healthyumi, Gogeonmi, Singeonmi, Jinhongmi, Jami, Jeungmi, and Annobeni. The pathogen inoculation experiment was conducted by following the method described above in the previous paragraph for the virulent pathogen. On the basis of symptoms, a disease severity index was established and categorized using a 0-to-5 scale. Categories were as follows: 0 = seedlings grew normally without visible symptoms; 1 = ~5% browning and cracking in vascular bundle length; 2 = ~25% browning and cracking, 3 = ~25% browning and cracking in vascular bundle length and yellowing of the bottom leaves; 4 = browning of the entire vascular and discoloration of all the leaves; 5 = seedling collapsed and died [49]. The final disease index (DI) was calculated using the following formula:

(2) $$\mathrm{DI}=\left[\frac{1\;n+2\;n+3\;n+4\;n+5\;n}{N}\right]\times \;100$$

where N is the total number of plants of each treatment and *n* is the number of plants in each category of disease severity [44]. Using this formula sweetpotato plants were categorized as high resistant—disease index ranged from 0.0 to 1.0; moderately resistant—1.1 to 2.5; and susceptible—>2.6.

## Figures and Tables

**Table 1:** Incidence of Fusarium wilt in different locations in Korea from 2015 to 2017.

Number of Locations	Number of Fields	Area (ha)	Disease Incidence Rate (%)	Year
8	233	249.3	17.9	2015
8	223	470.9	5.9	2016
6	182	501.0	8.3	2017

**Table 2:** List of *F. oxysporum* isolates sequenced in the present study and their GenBank information along with their relatives to construct a phylogenetic tree.

Species	Origin	Host	Isolates/Strain	GenBank Accession Number	
				ITS	EF
*Fusarium armeniacum*	USA	Horse eye	NRRL 43641	GQ505462	GQ505430
*F. asiaticum*	USA	*Triticum aestivum*	NRRL 26156^T^	NR_121320	AF212452
*F. bigoniae*	Germany	*Begonia hiemalis*	CBS 452.97^T^	NR_111864	KC514054
*F. bactridioides*	USA	*Pinus leiophylla*	CBS 100057^T^	NR_120262	KC514053
*F. cerealis*	Argentina	Barley grains	MAFF 241212	AB820717	AB820701
*F. circinatum*	USA	*Pinus radiata*	CBS 405.97^T^	NR_120263	KM231943
*F. commune*	USA	*Lycopersicon esculentum*	NRRL 28387	NA^a^	HM057338
*F. concentricum*	Costa Rica	*Musa sapientum*	NRRL 25181^T^	NR_111886	AF333935
*F. denticulatum*	USA	*Ipomoea batatas*	BBA67772	KR909405	KR909385
*F. denticulatum*	USA	*Ipomoea batatas*	CBS 407.97	NR_138359	AF160269
*F. equiseti*	Germany	Soil	NRRL 26419^T^	NR_121457	GQ505599
*F. graminearum*	USA	*Triticum aestivum*	CBS 130917	JX162342	JX118950
*F. guttiformae*	S. America	*Ananas comosus*	CBS 409.97	NA	KC514066
*F. incarnatum*	NA	Human skin	CBS 133024	KF255449	KF255493
*F. kyushuense*	Japan	NA	MAFF 237645	AB587019	AB674296
*F. mexicanum*	Mexico	*Mangifera indica*	MXMIC-686	-	KM823584
*F. miscanthi*	Belgium	*Miscanthus giganteus*	MUCL53238	-	HQ683752
*F. napiforme*	South Africa	*Pennisetum typhoides*	CBS 673.94	KR071649	KR071715
*F. oxysporum*	Poland	NA	CBS 129.24	DQ453704	
*F. oxysporum*	NA	Human skin	CBS 133023	KF255448	KF255492
*F. oxysporum*	NA	NA	CBS 140424	KT794176	KT794174
*F. oxysporum*	Mexico	*Capsicum annuum*	CPO 3.011	NA	KR935895
*F. oxysporum* f. sp. *chrysanthemi*	Korea	*Chrysanthemum indicum*	ATCC 52422	NA	DQ452426
*F. oxysporum* f. sp. *dianthi*	Spain	*Dianthus caryophyllus*	Fod 275	NA	KJ433854
*F. oxysporum* f. sp. *radicis-lycopersici*	USA	*Lycopersicon esculentum*	CL-06202	NA	HM057332
*F. oxysporum*	Korea	Wilt, *I. batatas*	SPL15020	KY508368	KY508356
*F. oxysporum*	Korea	Wilt, *I. batatas*	SPL15023	KY508366	KY508354
*F. oxysporum*	Korea	Wilt, *I. batatas*	SPL15037	KY508365	KY508353
*F. oxysporum*	Korea	Roots, *I. batatas*	SPL16046	KY508358	KY508346
*F. oxysporum*	Korea	Roots, *I. batatas*	SPL16048	KY508357	KY508345
*F. oxysporum*	Korea	Wilt, *I. batatas*	SPL18018	-	-
*F. oxysporum*	Korea	Wilt, *I. batatas*	SPL16019	-	-
*F. penzigii*	UK	*Fagus sylvatica*	CBS 317.34^T^	NA	EU926324
*F. poae*	Japan	*Glycine max*	MAFF 305947	AB587024	AB674302
*F. polyphialidicum*	Africa	Plant debris	CBS 676.94	X94172	KR071774
*F. temperatum*	Belgium	*Zea mays*	EFA313A	KC179826	KC179824
*F. thapsinum*	Africa	*Sorghum bicolor*	NRRL 22045	U34560	AF160270
*F. solani*	Australia	Bark	CBS 102429	KM231808	KM231936
*F. solani*	Sudan	*Vicia faba*	NRRL 52715	JF740912	DQ247657
*F. subglutinans*	USA	*Zea mays*	CBS 215.76	NA	KC514067
*F. verticillioides*	Germany	*Zea mays*	NRRL 22172	U34555	AF160262

‘T’ indicates type strain and ‘a’ indicates not available data.

## Supplementary Materials

The following are available online at https://www.mdpi.com/2223-7747/9/4/497/s1, Table S1: Cultural and morphological characteristics (conidial characteristics) of the isolates obtained in the present study.
