# Supplementary material for: Occurrence of Sweetpotato (Ipomoea batatas) Wilt and Surface Rot Disease and Determining Resistance of Selected Varieties to the Pathogen in Korea
Source: Plants (Basel). 2020 Apr 13;9(4):497. doi: 10.3390/plants9040497 (PMC7238964; doi:10.3390/plants9040497)
Supplement: Supplementary file 1 [file plants-09-00497-s001.pdf]

Table S1. Cultural and morphological characteristics (conidial characteristics) of the isolates obtained in the present study.

Isolate Number	Conidial Size (μm at 25 °C)		Conidial Characteristics			
	Macro	Micro	Macro	Septa	Micro	Septa
SPL15020	10.8~(25.9)~35.3×2.4~(3.7)~5.2	3.1~(5.7)~7.7×1.4~(2.5)~3.4	Slightly curved	Usually 3	Elliptical or cylindrical	0
SPL15023	13.0~(30.9)~39.5×2.1~(3.5)~4.4	3.8~(5.9)~7.7×1.7~(2.7)~3.7	Slightly curved	Usually 3	Elliptical or cylindrical	0
SPL15037	15.2~(28.2)~41.9×2.3~(3.2)~4.5	3.6~(6.0)~7.7×1.6~(2.2)~2.9	Slightly curved	Usually 3	Elliptical or cylindrical	0
SPL16046	11.1~(24.7)~34.6×4.9~(5.7)~6.9	4.3~(6.4)~8.3×2.2~(3.0)~3.7	Slightly curved	Usually 3	Elliptical or cylindrical	0
SPL16048	12.7~(21.5)~28.4×2.1~(3.9)~5.6	4.5~(6.3)~7.9×1.8~(2.6)~3.8	Slightly curved	Usually 3	Elliptical or cylindrical	0
SPL18019	14.0~(25.4)~36.8×2.6~(3.9)~5.3	4.3~(7.3)~10.4×2.2~(2.9)~4.1	Slightly curved	Usually 3	Elliptical or cylindrical	0
*F. oxysporum**	32.0~41.3×3.5~8.9	5.2~10.1×2.2~4.7	Sickle-shaped	3 to 4	Oval-ellipsoid to cylindrical	0
